# A Retrospective, Nationwide, Multicenter Study on Diagnosis and Treatment Outcome of Pediatric Optic Pathway/Hypothalamic Gliomas Including Analysis of Risk Factors for Progression After Systemic Anticancer Therapy

**DOI:** 10.3390/cancers17050716

**Published:** 2025-02-20

**Authors:** Carlien A. M. Bennebroek, Judith van Zwol, Maartje C. Montauban van Swijndregt, Giorgio L. Porro, Rianne Oostenbrink, Anne T. M. Dittrich, Jan W. Pott, Lisethe Meijer, Etienne J. M. Janssen, Sylvia Klinkenberg, Noel J. Bauer, Irene C. Notting, Maria M. van Genderen, Michael W. Tanck, Pim de Graaf, Peerooz Saeed, Antoinette Y. N. Schouten-van Meeteren

**Affiliations:** 1 Department of Ophthalmology, Amsterdam UMC, University of Amsterdam, 1012 WP Amsterdam, The Netherlands; judith.vanzwol@amsterdamumc.nl (J.v.Z.); m.c.montaubanvanswijndregt@amsterdamumc.nl (M.C.M.v.S.); p.saeed@amsterdamumc.nl (P.S.); 2 Cancer Center Amsterdam, Cancer Treatment and Quality of Life, 1066 CX Amsterdam, The Netherlands; 3 Department of Ophthalmology, Utrecht UMC, 3584 CX Utrecht, The Netherlands; g.porro@umcutrecht.nl (G.L.P.); mvgenderen@bartimeus.nl (M.M.v.G.); 4 ENCORE-NF1 Center, Department of General Pediatrics, Erasmus MC, 3015 GD Rotterdam, The Netherlands; r.oostenbrink@erasmusmc.nl; 5 Department of Pediatrics, Radboud University Medical Center, Amalia Children’s Hospital, 6525 GA Nijmegen, The Netherlands; anne.dittrich@radboudumc.nl; 6 Department of Ophthalmology, University Medical Center Groningen, University of Groningen, 9712 CP Groningen, The Netherlands; j.w.r.pott@umcg.nl; 7 Department of Neuro-Oncology, Princess Máxima Center for Pediatric Oncology, 3584 CS Utrecht, The Netherlands; l.meijer-13@prinsesmaximacentrum.nl (L.M.); a.y.n.schouten@prinsesmaximacentrum.nl (A.Y.N.S.-v.M.); 8 Department of Pediatrics, MosaKids Children’s Hospital, Maastricht University Medical Center, 6229 HX Maastricht, The Netherlands; etienne.janssen@mumc.nl (E.J.M.J.); s.klinkenberg@mumc.nl (S.K.); 9 Department of Ophthalmology, Maastricht University Medical Center, 6229 HX Maastricht, The Netherlands; n.bauer@mumc.nl; 10 Department of Ophthalmology, Leids University Medical Center, 2333 ZA Leiden, The Netherlands; i.c.notting@lumc.nl; 11 Diagnostic Center for Complex Visual Disorders, Bartiméus, 3703 AJ Zeist, The Netherlands; 12 Department of Epidemiology and Data Science, Amsterdam UMC, University of Amsterdam, 1012 WP Amsterdam, The Netherlands; m.w.tanck@amsterdamumc.nl; 13 Department of Radiology and Nuclear Medicine, Amsterdam UMC, Vrije Universiteit Amsterdam, 1081 HV Amsterdam, The Netherlands; p.degraaf@amsterdamumc.nl; 14 Cancer Center Amsterdam, Imaging and Biomarkers, 1012 WP Amsterdam, The Netherlands

**Keywords:** child, low-grade glioma, optic pathway glioma, neurofibromatosis type 1, progression, survival, prognostic factor, systemic anticancer therapy, chemotherapy, radiotherapy

## Abstract

**Background:** The current standard therapy for pediatric optic pathway/hypothalamic glioma (OPHG) is systemic anticancer therapy (SAT) over surgery and radiotherapy. Nevertheless, recurrent radiological or clinical tumor progression after SAT forms a considerable challenge. Sporadic OPHGs are considered to have a higher tendency toward progression after first-line systemic anticancer therapy (SAT) compared to neurofibromatosis type-1-associated (NF1) OPHGs. **Methods:** The objective of this study was to conduct a national retrospective cohort analysis of children who received various treatments for a progressive OPHG, involving the hypothalamus and/or chiasm and/or optic radiations. The study aimed to examine the differences in clinical course and the range of treatment modalities applied to both sporadic and NF1-associated OPHGs between 1995 and 2020. Additionally, we sought to identify risk factors for 3- and 5-year progression following first- and second-order SAT. **Results:** In total, 136 children received treatment, of whom 49 of 136 (36.0%) had NF1. Within a median of 7.5 years (range: 0.1–23.8 years) of follow-up, sporadic OPHGs received more treatments compared to NF1-associated OPHGs (median of 2 (range: 1–8) vs. median of 1 (range: 1–7) (*p* < 0.01)). Nine children with sporadic OPHGs (6.6%) died. Of 112 children (82.4%) receiving SAT, 92% received combined first-line vincristine and carboplatin. These children had a 3- and 5-year progression-free survival of 61.8% (95% CI: 51.0–72.6%) and 48.4% (95% CI: 38.0–58.8%), respectively. Sporadic OPHGs had a higher rate of second progression (*p* < 0.01). Starting first-line vincristine and carboplatin at an age below one year was the only independent risk factor for progression. **Conclusions:** In this national historic cohort of pediatric OPHGs, four out of five children received SAT. Sporadic OPHGs received a higher number of various SATs compared to NF1-associated OPHGs, but the sporadic appearance of OPHGs was not an independent risk factor for progression after combined vincristine and carboplatin, as ‘age below one year at the start’ was the only factor.

## Simple Summary

Systemic anticancer therapy (SAT) is the preferred treatment approach for progressive pediatric optic pathway/hypothalamic gliomas (OPHGs) to delay or avoid the need for surgery and radiotherapy. Nevertheless, recurrent progression appears in 30–50% of children. This national cohort study examines the variation in treatment received by children with an OPHG between 1995 and 2020. The study focuses on the effectiveness of SAT for both sporadic and neurofibromatosis type-1-associated (NF1) OPHGs. Risk factors for progression after first-line treatment with vincristine and carboplatin were identified. The results show that sporadic OPHGs received more treatments than NF1-associated OPHGs and had a higher rate of progression following first-line vincristine/carboplatin therapy. Furthermore, children initiating first-line SAT before the age of one year had an increased risk of progression of the OPHG. These findings underscore the need for personalized treatment strategies, especially for young children, and could guide future research on optimizing OPHG management in pediatric patients.

## 1. Introduction

Pediatric optic pathway/hypothalamic glioma (OPHG) is a subset of low-grade glioma (LGG), primarily localized in chiasm, the optic tract with or without optic nerve involvement. OPHGs represent approximately 2–5% of all pediatric intracranial tumors and are histologically classified as pilocytic astrocytomas (PAs) in 85–90% [1,2]. These tumors are associated with neurofibromatosis type 1 (NF1) in 30–50% of children. While OPHGs are generally slow growing, their progression can lead to significant morbidity, including the loss of visual functions, neurological deficits, and, in some cases, death.

Progressive OPHGs requiring intervention are generally treated with systemic anticancer therapy (SAT), represented by various chemotherapy strategies, anti-VEGF- and MAP-kinase-directed therapy, because surgery poses a high risk of damaging adjacent critical brain structures. Also, both surgery and radiotherapy can lead to severe cognitive, visual, and endocrine impairment [3,4]. Additionally, radiotherapy can result in cerebral vasculopathy [5,6,7] or secondary tumors in patients with NF1 [8], which emphasizes the importance of maximally delaying radiotherapy in young children.

Considering the high overall survival (OS) rate of 90–100% [9], the use of SAT requires ongoing balancing between effectiveness and toxicity to minimize long-term sequelae resulting from both disease and therapy. Over the last four decades, combined vincristine and carboplatinhas become the most frequently applied standard of care for first-line SAT [10,11], with vinblastine is considered the first-line standard of SAT in some countries [12]. However, no global consensus exists on the use of successive SAT [13]. The current experience with available SAT strategies is characterized by progression after both first-line and successive treatment lines, which places a long-term burden on both children and their parents [10,12,14]. Recently, targeted therapy of the Ras/Raf/4 signaling pathways has revealed promising outcomes in effectively reducing tumor volume and minimizing the rate of progression [15,16], leading to changes in subsequent SAT strategies.

Studies investigating risk factors for progression following initial SAT for an OPHG have concluded that children starting treatment below the age of one year are at an increased risk of recurrent progression [10,14]. Additionally, sporadic OPHGs (not associated with NF1) are suggested to have a more aggressive disease course compared to NF1-associated OPHGs, leading to earlier progression after SAT [12,14]. This evidence is supported by studies on LGGs that include various anatomical locations [17] or studies on diverse LGG locations including separate analyses on OPHGs [18]. The former do not provide a fair representation of treatment outcomes specifically for OPHGs, which is reflected in studies comparing outcome for diverse anatomic locations of LGGs, as 5- and 10-year progression-free survival (PFS) rates are significantly lower for OPHGs compared to LGGs in other cerebral locations [2,17,19]. Consequently, there is an ongoing need for more comprehensive data to assess treatment outcomes based on NF1 status and to work toward individualized treatment strategies for children at risk of a more aggressive course.

In this retrospective national cohort study, we present an overview of diagnosed and treated OPHGs, including focus on comparing progression, survival outcomes, and risk factors for progression between NF1-associated and sporadic OPHGs across various treatments.

## 2. Material and Methods

### 2.1. Study Population

Data were retrospectively obtained from children aged 0–17 years who were diagnosed with an OPHG, involving the hypothalamus and/or chiasm and/or optic radiations, between January 1995 and December 2018, as well as from children who were treated for progressive OPHGs between January 1995 and December 2020. Data were collected from children who were registered in the national database of the Dutch Childhood Oncology Group (DCOG) between January 2003 and December 2018. Additionally, existing local databases in the pediatric oncology, ophthalmology, and neurology departments of all participating centers were reviewed to include patients from January 1995 to December 2018. Patients with an isolated optic nerve glioma, both uni- and bilateral, were excluded, as these were published separately [20]. Patients were treated in one of the eight university medical centers in the Netherlands or at the national pediatric oncology center (Princess Máxima Center). The study included both OPHGs associated with NF1 and sporadic OPHGs. Data were included from all children who received various treatments for a progressive OPHG located within the chiasm and/or optic tract and/or involvement of the optic nerve(s), with or without hypothalamic involvement. To represent the complete national cohort of diagnosed pediatric OPHGs, baseline data were collected for patients who did not receive treatment.

The study received approval from the DCOG and the ethics committees of all university medical centers, the Princess Máxima Center, and two visual rehabilitation centers waived approval according to the Medical Research Involving Human Subject Act. Informed consent was obtained from patients (or their parents/guardians) registered with the DCOG and UMC Utrecht. An opt-out procedure was offered to patients registered in the local databases of the Amsterdam UMC. Other centers granted permission to use anonymized patient data.

### 2.2. Data Collection

Demographic information, tumor-related characteristics, and the sequence of treatment modalities were extracted from medical records and compared between NF1-associated and sporadic OPHGs. The diagnosis of OPHGs was based on biopsy or, in the case of unequivocal neuroradiological findings, on orbital (T2-weighted, STIR, or contrast-enhanced fat-suppressed T1-weighted) or brain (T2, FLAIR or contrast-enhanced T1-weighted) MRI. The NF1 status was assessed by clinical criteria substantiated by DNA analysis, when available.

In the Netherlands, until 2017, the individual indication and choice of treatment for progressive OPHGs were determined by local academic treatment teams. This process was subsequently centralized with the establishment of the national pediatric oncology center from 2018 on. We defined ‘progression’ as the start of first-line or successive treatment or as significant clinical deterioration (visual and/or neurological but not endocrinological functions) or radiological progression defined by the local multidisciplinary team, but in the absence of further treatment. First-line SAT, mostly represented by a combination of vincristine and carboplatin, was generally administered, according to the SIOPe LGG study protocol [21], represented by a standard induction regimen involving ten weekly administrations of vincristine at a dose of 1.5 mg/m^2^ via intravenous bolus, along with four single doses of carboplatin at 550 mg/m^2^ as a 1-hour intravenous infusion, administered at 3-week intervals. This was followed by three cycles of concurrent VC treatment at 4-week intervals (doses were adjusted by weight for children below the age of 1 year and/or 10 kg). Total chemotherapy treatment lasted 18 months [22].

In the case of a preterm change in the SAT due to side effects, this switch was not considered as successive SAT. The anatomic location of the OPHG was evaluated according to the modified Dodge classification (MDC) [23] by a 14-year-experienced neuroradiologist (PG).

Three- and five-year progression-free survival (PFS) and OS analyses were performed following the start of first-line vincristine and carboplatin, as this combination accounted for more than 90% of first-line SAT and all second-line SAT. Risk factors for progression after the start of SAT were assessed at three and five years after the start of first-line vincristine and carboplatin, as well as all second-line SAT.

### 2.3. Statistical Analysis

Continuous data are presented as the mean and standard deviation (normal distribution) or median and range (non-normal distribution) and categorical data by frequency and percentage. Regarding the NF1 status, the between-group differences were examined using the Student’s *t*-test for normally distributed continuous data, the Mann–Whitney U test for non-normally distributed continuous data, and the χ^2^ test or Fisher’s exact test for categorical data. The three- and five-year PFS and OS were calculated using the Kaplan–Meier method, defining progression as the date of start of the successive therapy or the date of defined progression with no subsequent treatment. Patients were censored in the case of death or loss to follow-up. Stratified comparison of the PFS distribution was performed using log-rank analysis (*p* < 0.05).

Cox proportional hazards regression was used for both univariable (*p* < 0.05) and multivariable analyses (*p* < 0.2 in the univariable analysis) to identify prognostic factors for 3- and 5-year PFS (*p* < 0.05). The analyzed variables were NF1 status, age at the start of treatment, and the anatomic location (MDC) of OPHGs. Statistical analyses were conducted using SPSS software for Windows (version 26.0.0.1, SPSS Inc., Chicago, IL, USA).

## 3. Results

Between 1995 and 2018, 197 children were diagnosed with an OPHG (see Table 1). Two patients were excluded because their parents refused data collection. Fifty-nine (29.9%) patients did not receive treatment, 91.5% of them had NF1. They were diagnosed at a median age of 5.8 years (range: 0.2–16.7 years) and followed for a median of 9.1 years (range: 0.1–23.8 years). Within the NF1 population, no significant differences were observed in sex, age at diagnosis, or anatomic location of the OPHG between patients who received various treatments and those who did not (*p* = 0.27, *p* = 0.10, and *p* = 0.09). Within the population of untreated patients, one child with NF1 was diagnosed at the age of four months and did not receive treatment before dying.

### 3.1. Baseline Characteristics

A total of 136 children (69.7%) who received treatment were included in this study. Among them, 36.0% had NF1 and 45.6% were male. Children were diagnosed at a median age of 4.6 years (range: 0.3–16.5 years). Sporadic OPHGs were diagnosed at a younger age (median: 3.8 years) compared to NF1-associated OPHGs (median: 5.4 years) (*p* < 0.01). Treatment was initiated at a median age of 5.4 years (range: 0.3–16.5 years) and was started at a younger age for sporadic OPHGs (median: 4.2 years) compared to NF1-associated OPHGs (median: 7.3 years) (*p* < 0.01). Treatment for sporadic OPHGs started at a shorter interval after diagnosis compared to NF1-associated OPHGs (*p* < 0.01). Ninety-two children (67.7%) started with therapy shortly after diagnosis. The median follow-up period was 7.5 years (range: 0.1–23.8 years), with no difference based on the NF1 status. Diffuse OPHGs, extending along the optic tract (MDC stages: (1–)2–3–(4)), were present in 64.0% of cases, and hypothalamic involvement was present in 62.5%. Both features were more common in sporadic OPHGs (*p* < 0.01). Initial treatment was started based on abnormalities in visual function in 76.0%, while neurological and/or endocrine symptoms were present in 42.1%. Histopathological analysis was available for 75 OPHGs (55.1%), of which 84.0% were pilocytic astrocytomas. The baseline characteristics of the study cohort are summarized in Table 2.

### 3.2. Treatment

**Figure 1:**
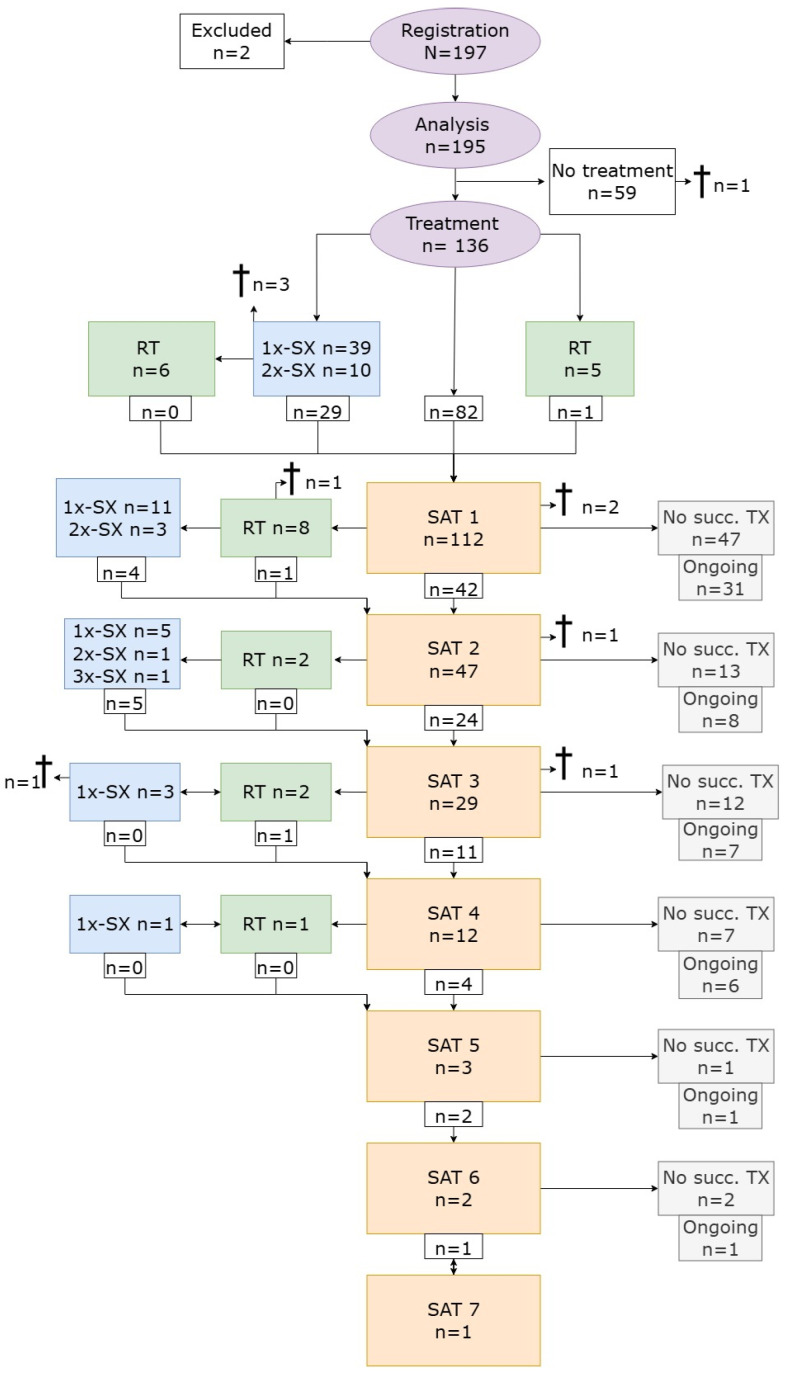
Flowchart of various (successive) treatments for progressive pediatric OPHGs. RT: radiotherapy; SAT: systemic anticancer therapy; Succ.: successive; SX: surgery; TX: therapy. † Number of patients that died. Subscript: In the case of a preterm change in the SAT due to side effects, a switch in the SAT was not considered as successive SAT.

The population received a median of two various treatments (range: 1–8). Sporadic OPHGs received a median of two treatments, compared to one in NF1-associated OPHGs (*p* < 0.01). A flowchart representing the sequence of the various treatments is presented in Figure 1. In total, 37.5% received a single episode of first-line treatment only, which was more common in NF1-associated OPHGs (58.8%) compared to sporadic OPHGs (41.2%) (*p* < 0.01). Thirty children (22.1%) received more than three different treatments, among whom 83.3% had a sporadic OPHG.

Initial resection was performed in 49 children (36.0%), followed by successive therapy in 37 children (75.5%) within a median of 0.3 years (range: 0.0–7.2 years). In total, nine children (6.6%) who received treatment died, seven of them because of progression of the OPHG. One patient died because of infectious meningitis and one died by suicide. All patients had a sporadic OPHG. One patient died within one month after diagnosis at the age of four months because of the progression of the OPHG. Treatment was not initiated at the request of the parents, because of rapid progression, discomfort, and absence of visual functions in the child. The NF1 status was not determined, but no clinical signs fitting NF1 were present.

### 3.3. Systemic Anticancer Therapy

**Figure 2:**
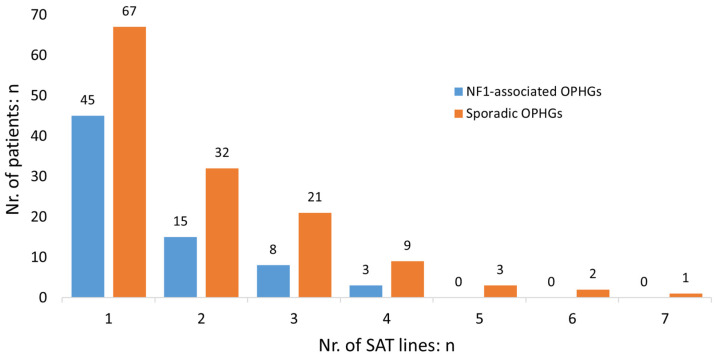
Successive number of treatment lines of systemic anticancer therapy (SAT) (n = 112): a comparison of sporadic and NF1-associated OPHGs. NF1: neurofibromatosis type 1; Nr: number; SAT: systemic anticancer therapy; sporadic: OPHG not associated with NF1.

First-line SAT was administered to 112 patients (82.4%). Seventy-five children (67.0%) started within three months after the diagnosis of OP. Thirteen children (11.6%) started SAT within three months after surgical resection. Combined vincristine and carboplatin was administered in 103 children (92.0%), of whom 46 children (44.7%) developed an allergic reaction to carboplatin, resulting in 23 children (50%) switching SAT. Second-line SAT was administered to 47 children (42.0%), consisting of vinblastine monotherapy in 59.6% and a combination of bevacizumab and irinotecan in 19.1%. Children with sporadic OPHGs received a greater number of SAT lines compared to children with NF1-associated OPHGs (*p* < 0.01) (see Figure 2). An overview of the different types and combinations of successive SAT lines is provided in Table 3.

**Figure 3:**
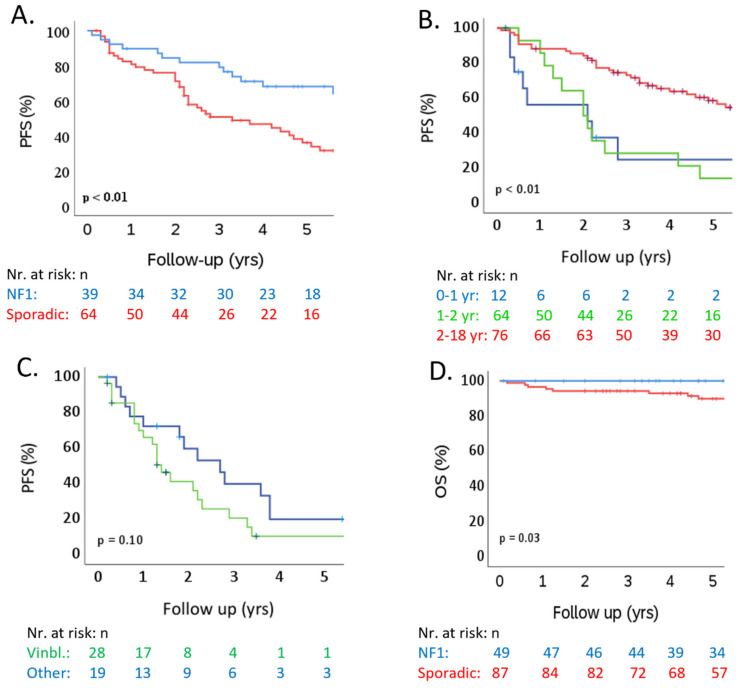
Kaplan–Meier curves of PFS since the start of first-line vincristine/carboplatin and various second-line SATs (n = 103), as well as the OS of the whole cohort that received various treatments. (**A**) PFS after first-line vincristine/carboplatin: a comparison of NF1-associated and sporadic OPHGs. (**B**) PFS after first-line vincristine/carboplatin (n = 103): a comparison of age categories (0–<1 yr vs. 1 to <2 yr vs. 2–<18 yr). (**C**) PFS after second-line SAT (n = 47: a comparison of vinblastine and other various second-line SAT (see Table 3)). (**D**) OS of the total cohort (n = 136) after various treatments: a comparison of NF1-associated and sporadic OPHGs (n = 136). NF1: neurofibromatosis type 1; Nr: number; OS: overall survival; PFS: progression-free survival; SAT: systemic anticancer therapy; sporadic: no association with NF1; VCR/CBDCA: vincristine/carboplatin; Vinbl.: vinblastine; yr(s): year(s).

The 3- and 5-year PFS rates following first-line treatment with vincristine and carboplatin were 61.8% (95% CI: 51.0–72.6%) and 48.4% (95% CI: 38.0–58.8%). The progression rate was higher for sporadic OPHGs compared to NF1-associated OPHGs (log-rank test: *p* < 0.01) and varied across the four different age categories (log-rank test: *p* < 0.01). The 3- and 5-year OS rates were 95.5% (95% CI: 91.9–99.1%) and 93.5% (95% CI: 88.9–98.1%). Regarding second-line SAT (n = 47), the 3- and 5-year PFS rates were 28.6% (95% CI: 13.8–43.4%) and 13.1% (95% CI: 1.5–24.7%). The rate of progression did not differ between sporadic and NF1-associated OPHGs (log-rank test: *p* = 0.22). Additionally, the rate of progression did not differ between second-line vinblastine and other second-line SAT combinations (*p* = 0.10). The Kaplan–Meier curves of PFS and OS for both first-line vincristine and carboplatin and second-line SAT are presented in Figure 3.

### 3.4. Predictive Factors for Progression After First-Line SAT

Uni- and multivariate analyses of risk factors for progression identified at an age younger than 1 year at the start of vincristine and carboplatin as an independent predictive factor for both 3- and 5-year PFS (hazard ratio (HR): 2.8, 95% CI: 1.3–6.1, *p* < 0.01))/(HR: 2.4, 95% CI: 1.2–4.8, *p* = 0.02). Sporadic OPHGs (3-year PFS: HR: 2.3, 95% CI: 1.3–4.1, *p* < 0.01)/(5-year PFS: HR: 1.6, 95% CI: 1.2–4.8, *p* < 0.01) and age >1–≤2 yr at the start of therapy (3-year PFS: HR: 2.5, 95% CI: 1.3–4.9, *p* < 0.01)/(5-year PFS: HR: 2.3, 95% CI: 1.2–4.2, *p* = 0.01) were identified as dependent risk factors for progression in univariable analysis (see Table 4). As no patient within the groups of children aged under two years at the start of therapy (0 to <1 and 1 to <2 years) had NF1, a sensitivity analysis was performed within the population of children aged 2 to <18 years using the variables of NF1 status and MDC location of the OPHGs. No (in-)dependent prognostic factor was identified for the population of this age category.

The analysis of second-line SAT, including second-line vinblastine monotherapy, did not identify any (in-)dependent prognostic factors for 3- or 5-year PFS.

### 3.5. Radiotherapy

Twenty-four children (17.6%) received RT at a median age of 10.0 years (range: 4.7–18.3 years). Five children (20.8%) received RT as an initial treatment, two of whom had NF1; all five were treated between 2002 and 2004. Nineteen children received RT as successive treatment, three of whom had NF1. In 87.5% of children, radiotherapy was initiated before 2014.

The patients received a median dose of 54.0 Gray (range: 45.0–57.6 Gray) and were followed for a median of 15.3 years (range: 3.8–23.4 years). The 3- and 5-year PFS rates were both 86.5% (95% CI: 72.1–100.0%), and the 10-year PFS was 80.0% (95% CI: 63.6–98.2%). No difference in PFS was observed between NF1-associated and sporadic OPHGs. No patient died during follow-up.

## 4. Discussion

This national cohort study provides a historical overview of the number and diversity of treatments applied to children with progressive OPHGs. NF1-associated OPHGs accounted for 36.0% of cases. These OPHGs were diagnosed and treatment was started at an older age than for sporadic OPHGs and received a lower total number of treatments. Among the population that received first-line vincristine and carboplatin, children with a sporadic OPHG who started this treatment below the age of one year, had an increased risk of progression.

To our knowledge, no nationwide historic cohort study has been published on pediatric OPHGs so far. One study presented monocenter data on the natural history of both sporadic and NF1-associated OPHGs [24] and two on NF1-associated OPHGs only [25,26]. All studies included both isolated optic nerve gliomas (ONGs) (8.5–48.1%) and other OPHGs in the overall analysis. We published a separate report on these entities, as progression rates differ among ONGs and OPHGs [20]. Nicolin et al. [24] reported on NF1-association in 58.6% of the diagnosed OPHGs, including treatment in 23.1% of NF1-associated and 92.7% of sporadic OPHGs. NF1-associated OPHGs were diagnosed at a younger age compared to sporadic OPHGs (*p* < 0.01); no data were available on the age at the start of treatment. Data extraction for pure OPHGs (excluding ONGs) could not be performed [24].

Trevisson et al. [26] and Listernick et al. [25] reported incidences of NF1-associated OPHGs, including ONGs, at screening rates of 12.6% and 18.8%, respectively, compared to 7.1% and 11.2% without ONGs. MR imaging was performed for 44.7% and 100.0%. Treatment was initiated in 15.3% (OPHG and ONG) and 6.1% (OPHG only) cases. In our study, 47.6% of the NF1-associated OPHGs and 94.6% of sporadic OPHGs received treatment. Comparing the treatment rates for NF1-associated OPHGs across these studies, we suspect that the high treatment rate in the present study, may be caused by an underreporting of NF1-associated OPHGs that were discovered at screening and did not require treatment, as these patients may have been registered incompletely in the Netherlands. Likewise, MRI examination is performed only in the case of a clinically suspected OPHGs. Both factors may have contributed to a selection bias.

Similar to previous studies, in the present study, no clinical differences (sex/age at diagnosis/MDC status) were observed between the OPHGs that received treatment or did not (both NF1-associated and sporadic OPHGs).

Table 1 shows the 5-year incidence of the number of children that were diagnosed and started treatment, suggesting an increase in the number of OPHGs receiving treatment since 2010. This can be explained by an inclusion bias in the years prior to the start of the national registration by the DCOG and, potentially, by a true increase in the treatment rate due to heightened awareness of clinical treatment indications and growing confidence in the effects of SAT. To provide insight into the differences in local treatment approaches, we investigated the rate of initial radiotherapy or surgery per institute, but no clear differences in the initial local approach could be identified. We did not perform an analysis of a possible shift in SAT strategy because of the small number of patients receiving alternative treatments compared to the predominance of vincristine and carboplatin as first-line therapies.

With the increasing use of new SAT modalities in recent decades, prolonged treatment with successive SAT strategies for recurrent progressive disease has postponed the use of radiotherapy, as is shown in our cohort with a maximum of seven successive SAT modalities. In this study, targeted therapy was administered in only seven patients as successive treatment. Regarding targeted therapy, there are early promising data on the efficacy [15], as radiological response and progression rates appear favorable, but phase three studies are ongoing [16]. Likewise, the discussion of the prioritization of targeted therapy requires a comparison with the current first-line SAT modalities in the subgroup solely consisting of OPHGs (not LGGs at diverse anatomical locations).

The two largest studies evaluating combined first-line vincristine and carboplatin (and etoposide in 1 treatment arm) [22] for LGG show 5-year PFS rates of 43.5% (n = 169) [22] and 60.5% (n = 123) [10]. In the first study, NF1-association was present in 29.3% and the median age at the start of SAT was 4.3 years in the total population (n = 497 LGG). In the second study, the NF1 incidence could not be extracted, and the median age at the start of SAT was 2.9 years. Kotch et al. reported on 103 patients with NF1 who received vincristine and carboplatin in 75.9%, and 26% had an ONG. Children started treatment at the age of 3.3 years [27]. Forty percent of the total cohort showed progression within five years. In our study, the 5-year PFS rate was 48.4% (n = 103), 37.9% had NF1, and the median age at the start of SAT was 5.4 years.

First-line treatment with vinblastine for LGGs (n = 55) showed a 5-year PFS rate of 53.2%. In 54.4%, LGGs were represented by OPHGs, but no separate analysis on PFS was available for OPHGs [12]. The French BBSFOP trial showed a 5-year PFS rate of 34.0%, NF1 was present in 27%, the median age at the start of SAT was 1.4 years [14].

Identifying factors that increase the risk of progression after first-line or successive therapy is essential given the rarity of pediatric OPHGs, the rate of progression, and the risk of impaired vision. Earlier studies on prognostic factors for progression after first-line SAT that focused solely on pediatric OPHGs, including both NF1-associated and sporadic OPHGs, identified several risk factors. These include children starting therapy before one year of age [10,14], non-pilocytic astrocytoma [10], sporadic appearance of OPHGs [14], and/or failure of an early response after first-line SAT [14] as independent risk factors for progression. The presence of diencephalic syndrome was considered a dependent prognostic factor for progression [10]. A multicenter study on NF1-associated OPHGs by Kotch et al. showed that children starting SAT below the age of two years and a posterior tumor location of the OPHG, including the optic tract, were both risk factors for progression at two and five years after the start of SAT [27]. This was the only available study in which children with NF1 started treatment under two years of age. Also, treatment failure with decreased survival in infants below one year was shown by Mirow et al. [28]. In the present study, children who started first-line vincristine/carboplatin treatment below the age of one were at increased risk of progression. However, in addition to the infants below one year of age, the initiation of SAT at the age of >1–≤2 yr was found to be a dependent risk factor (*p* < 0.01) and a near-significant independent risk factor (*p* = 0.09/0.10) for progression after 3 and 5 years. Further studies with larger cohorts are needed to determine whether the population younger than >1–≤2 yr years, or possible older age, have an increased risk of progression. The sporadic appearance of OPHGs was identified as a dependent risk factor for progression; however, all children below the age of two years in this treated cohort had a sporadic OPHG, which is similar to studies by Gnekow [10] and Laithier [14] et al.

Children requiring treatment at such an early age are known to be at risk for long-term severe visual impairment or blindness [29], which was also observed in our study (results to be published in the near future). These children require intensified monitoring and more effective treatment modalities.

The studies discussed include the largest available analyses of first-line progression and risk factors for progression. However, they all vary in baseline characteristics and the type of SAT used, making effective comparison challenging. None of these studies included molecular profiles or examined their subsequent effect on tumor growth and the treatment response for OPHGs. Such information is crucial for optimizing treatment for children at increased risk of progression and worse functional outcome, as currently implemented in the LOGGIC/Firefly-2 study [16].

The use of RT in the treatment of OPHGs requires careful consideration. Despite high rates of long-term tumor control (10 yr PFS: 66–89%) and survival (10 yr OS: 83–100%) [3,4,30,31], patients remain at risk for long-term morbidity [6]. The role of NF1 status as a risk factor remains controversial in the literature, as RT in children with NF1, when applied below the age of ten years, increases the risk of death due to cerebrovascular accidents and secondary malignant tumors [6]. Children with sporadic OPHGs are suggested to have a lower risk of long-term progression five years after RT [6] compared to those with NF1-associated OPHGs, although this is contradicted by another study [31]. In our study, the 10-year PFS was 80.0%, which did not differ between NF1-associated (n = 5) and sporadic OPHGs (n = 19). First-line RT was applied in five patients between 2002 and 2004, indicating that treatment teams developed a learning curve and began to avoid early RT in subsequent years. The majority of irradiated children (87.5%) received RT between 1995 and 2013, suggesting that the need for RT decreased with the increasing availability of various SAT modalities, like bevacizumab and the emerging MAPK-targeted therapy. In future studies, these modalities do necessitate a thorough discussion, particularly in regard to their toxicity profiles. For instance, combinations of bevacizumab with irinotecan are associated with an increased incidence of gastrointestinal toxicities [32]. In the case of poor tolerance, transitioning from combination therapy to single-agent bevacizumab may be necessary, although alternative drug options in third- or fourth-line treatments are increasingly limited. This cohort specifically has long time survivors where future questions on long-term safety can be addressed. Regarding RT, the current treatment guidelines recommend RT only in a limited number of cases, preferably using high-precision techniques [33].

### Strengths and Limitations

A key strength of this study is its nationwide inclusion of children who received various treatments for progressive OPHG. Selection bias was minimized by searching local databases in addition to obtaining data via the national registry (DCOG). This comprehensive approach helped ensure a broad representation of cases diagnosed with OPHG. However, the retrospective design of the study should be interpreted in the context of several limitations.

First, the data for this study were collected from medical records and evaluations over a 25-year period, which may have led to inconsistencies in data affecting the results. Regarding missing data, details on the clinical presentation at the start of therapy were incomplete, including ophthalmological examination and the presence of diencephalic syndrome, as well as information on the decision on the initiation of treatment, treatment evaluation including toxicity rates after SAT, and long-term systemic effects after RT. Data on tumor biology, which is currently considered essential in treatment considerations [34], were lacking for more than 80% of biopsies; therefore, this outcome was not presented in this study.

Second, treatment approaches may have varied locally as knowledge of treatment effects and available SAT modalities evolved, leading to changes in the choice of treatment strategy. Nevertheless, we were unable to recognize a trend in differences of approach.

Third, the Kaplan–Meijer analysis of second-line SAT involved six different SAT strategies, represented by vinblastine in the majority. As PFS rates did not differ between vinblastine and the other five SAT strategies (Figure 3C), we did not extend the analysis with a comparison of the baseline characteristics of patients or tumors, which may have varied in NF1 status, age, and MDC location.

Fourth, the treatment evaluation lacked a structured radiological response assessment [35] and reporting of the outcomes on long-term visual functions. Therefore, data on visual functions will be published separately in the near future.

## 5. Conclusions

This study provides a national overview of 25 years of diagnoses and treatment of pediatric OPHGs. Seven out of ten children required treatment. Sporadic OPHGs received a greater number of treatments and exhibited a higher rate of progression compared to NF1-associated OPHGs. Children below the age of one year who were treated with first-line vincristine and carboplatin were at risk of progression after three and five years. These findings highlight the need for stratified treatment strategies based on the OPHG subtype and patient age.

## Figures and Tables

**Table 1:** The annual and 5-year numbers of children who were diagnosed with an OPHG and the number of children who received various treatments.

	Diagnosis OPHG: n	Diagnosis OPHG/ yr	Start TX: n	Start Various TX/yr: n (%)
Total	195		136 (69.7)	
1995–1999	12	2.4	5	1 (41.7)
2000–2004	34	6.8	23	4.6 (67.6)
2005–2009	38	7.6	26	5.2 (68.4)
2010–2014	62	12.4	46	9.2 (74.2)
2015–2018	49	9.8	36	7.2 (73.5)

TX: therapy; yr; years.

**Table 2:** Baseline characteristics of pediatric patients treated for a progressive OPHG: a comparison of NF1-associated and sporadic OPHGs.

	Total Population	NF1 ass. OPHGs	Sporadic OPHGs
Study cohort: n (%)	136 (100.0)	49 (36.0)	87 (64.0)
Male	62 (45.6)	23 (46.9)	39 (44.8)
NF1: Clinical diagnosis	-	22 (44.9)	-
DNA diagnosis	-	27 (55.1)	-
Indication start of treatment: n (%)			
Clinical abnormalities	121 (89.0)	35 (71.4)	86 (98.9)
Ophthalmological ^1^	92 (76.0)	25 (71.4)	67 (77.0)
Neurological/endocrinological ^2^	51 (42.1)	15 (42.9)	36 (41.9)
Radiological progression ^3^	9 (6.8)	8 (18.6)	1 (1.1)
No data	6 (4.4)	6 (12.2)	0 (0.0)
Anatomic location: MDC			
MDC 2	13 (9.6)	2 (4.1)	11 (12.6)
MDC 1–2	36 (26.5)	14 (28.6)	22 (25.3)
MDC (1-)2–3-(4) ^4^	87 (64.0)	33 (67.3)	54 (62.1)
Hypothalamic involvement	85 (62.5)	27 (55.1)	58 (66.7)
LM metastases	11 (8.1)	0 (0.0)	11 (100.0)
Histopathology (73)			
Biopsy/resection	75 (55.1)	10 (20.4)	65 (74.7)
Pilocytic astrocytoma	63 (84.0)	7 (70.0)	56 (86.2)
Pilomyxoid astrocytoma	7 (9.3)	0 (0.0)	7 (10.8)
Astrocytoma gr. 1	2 (2.7)	1 (10.0)	1 (1.8)
Astrocytoma gr. 2	1 (1.3)	0 (0.0)	1 (1.8)
Inconclusive	2 (2.7)	2 (20.0)	0 (0.0)
Treatment: m (r)			
Age at diagnosis (yr): m (r)	4.6 (0.3–16.5)	5.4 (1.5–13.9)	3.8 (0.3–16.5)
Age at start TX (yr): m (r)	5.4 (0.3–16.5)	7.3 (2.3–16.3)	4.2 (0.3–16.5)
Diagnosis—start TX (yr): m (r)	0.1 (0.0–13.5)	0.6 (0.0–13.5)	0.1 (0.0–7.3)
Start TX—end FU (yr): m (r)	7.5 (0.1–23.8)	7.1 (0.1–22.3)	7.6 (0.17–23.8)
Nr. of TX: m (r)	2 (1–8)	1 (1–7)	2 (1–8)
First-line SAT (pts): n (%)	112 (82.4)	45 (91.8)	67 (77.0)
VCR/CBDCA	103 (92.0)	39 (86.7)	64 (96.5)
VCR/CBDCA/ETP	3 (2.7)	0 (0.0)	3 (4.5)
Vinblastine	4 (3.6)	4 (8.2)	0 (0.0)
BVZ/irinotecan	1 (0.9)	1 (20)	0 (0.0)
Temozolomide	1 (0.9)	1 (2.0)	0 (0.0)
First-line TX only (pts): n (%)	51 (37.5)	30 (61.2)	21 (24.1)
SAT	36 (70.6)	27 (90.0)	9 (42.9)
SX ^5^	10 (21.6)	1 (3.7))	9 (42.9)
RT	5 (3.6)	2 (7.4)	3 (14.3))
SX ^5^: first-line or succ.: n (%)	66 (48.5)	8 (16.3)	58 (66.7)
RT: first-line or succ.: n (%)	24 (17.6)	5 (10.2)	19 (21.8)
>3 TXs: n (%)	30 (22.1)	5 (10.2)	25 (28.7)

ass: Associated; BVZ: bevacizumab; CBDCA: carboplatin; ETP: etoposide; FU: follow-up; LM: lepto-meningeal; MDC: modified Dodge classification (stage 1: optic nerve involvement; stage 2: chiasm; stage 3: anterior optic tract; stage 4: posterior optic tract); M (r): median (range); NF1: neurofibromatosis type 1; Pts: patients; sporadic: no association to NF1; RT: radiotherapy; SAT: systemic anticancer therapy; Succ.: successive; SX: surgery; TX: therapy; VCR: vincristine; yr: years. ^1^ Ophthalmological symptoms include a decrease in best-corrected visual acuity or visual field and/or proptosis. ^2^ Neurological or endocrinological symptoms, with or without ophthalmological symptoms. ^3^ Radiological progression on MRI, no clinical data available, or no clinical abnormalities. ^4^ The MDC (1–)2–3–(4) stages include chiasmatic and anterior optic tract involvement with possible involvement of the optic nerve(s) and/or posterior tract. ^5^ Surgery was represented by partial or complete surgical resection but not by biopsy only.

**Table 3:** Types of SAT in 112 children administered for a progressive optic pathway glioma.

	SAT 1	Course: m (r) (mnths)	SAT 2	Course: m (r) (mnths)	SAT 3	Course: m (r) (mnths)	SAT 4	Course: m (r) (mnths)	SAT 5	Course: m (r) (mnths)	SAT 6	Course: m (r) (mnths)	SAT 7	Course m (r) (mnths)
Total pts.	112		47		29		12		3		2		1	
VCR/CBDCA														
NF1	39	18 (1–32)	1	16										
sporadic	64	18 (1–24)	5	12 (10–20)										
VCR/CBDCA/ETP														
NF1	0													
sporadic	3	18 (7–19)			1									
VCR														
NF1			1	2.0										
sporadic														
Vinblastine														
NF1	4	11 (11–14)	8	12 (2–12)	2	4 (2–5)	1	7						
sporadic	0		20	11 (2–21)	9	7 (2–12)								
BVZ/IRI														
NF1	1	2	4	12 (4–24)	5	13 (8–22)	1	12						
sporadic	0		5	11 (6–14)	6	11 (6–15)	7	12 (6–36)	1	13				
BVZ														
NF1														
sporadic					1	6			1	18	1	55	1	22
BVZ/Vinbl.														
NF1														
sporadic			2	10 (8–12)	1	3	1	9						
Trametinib														
NF1							1	8						
sporadic					2	19 (13–24)	1	4	1	18	1	4		
Temozolamide														
NF1	1	5	1	4										
sporadic					2	9 (6–11)								

BVZ: bevacizumab; CBDCA: carboplatin; ETP: etoposide; IRI: irinotecan; mnths: months; M (r): median (range); NF1: OPHG associated with neurofibromatosis type 1; pts: patients; SAT: systemic anticancer therapy; sporadic: OPHG with no association to NF1; VCR: vincristine; Vinbl: vinblastine.

**Table 4:** Uni- and multivariate analyses by Cox regression: prognostic factors for 3- and 5-year progression-free survival after the start of first-line vincristine and carboplatin.

PFS		Univariate: 3-Year			Multivariate 3-Year			Univariate: 5-Year			Multivariate 5-Year		
Variables	n	HR	95% CI	*p*	HR	95% CI	*p*	HR	95% CI	*p*	HR	95% CI	*p*
NF1-ass. OPHGsOPHGs	39	Ref.	-	-	Ref.	-	-	Ref.	-	-	Ref.	-	-
Sporadic OPHGs	64	2.3	1.3–4.1	<0.01	1.6	0.8–3.1	0.17	1.879	1.2–4.8	0.01	1.4	0.8–2.4	0.24
Age: 0–≤1 yr	12	3.4	1.7–6.9	<0.01	2.8	1.3–6.1	0.01	2.8	1.5–5.4	<0.01	2.3	1.2–4.8	0.02
Age: >1–≤2 yr	15	2.5	1.3–4.9	<0.01	1.9	0.9–4.2	0.09	2.3	1.2–4.2	0.01	1.8	0.9–3.5	0.10
Age: >2–≤18 yr	76	Ref.	-	-	Ref.	-	-	Ref.	-	-	Ref.	-	-
MDC 2	10	Ref	-	-	Ref.	-	-	Ref.	-	-	Ref.	-	-
MDC 1 + 2	30	0.5	0.2–1.1	0.08	0.8	0.3–2.1	0.68	0.5	0.2–1.0	0.04	0.7	0.3–1.6	0.37
MDC (1–2)−3–(4)	63	0.5	0.2–1.1	0.08	0.7	0.3–1.6	0.38	0.4	0.3–1.1	0.09	0.7	0.3–1.4	0.32

Ass.: associated; CI: confidence interval; HR: hazard ratio; MDC: modified Dodge classification (stage 1: concurrent optic nerve involvement; stage 2: chiasm; stage 3: anterior optic tract; stage 4: posterior optic tract); NF1: neurofibromatosis type 1; PFS: progression-free survival; sporadic: OPHGs not associated with neurofibromatosis type 1; yr: year.

## Data Availability

The data presented in this study are available upon request from the corresponding author. The data are not publicly available to protect patient privacy.

## Abbreviations

CI	Confidence interval
DCOG	Dutch Childhood Oncology Group
LGG	Low-grade glioma
MDC	Modified Dodge classification
NF1	Neurofibromatosis type 1
ONG	Isolated optic nerve glioma
OPHG	Optic pathway/hypothalamic glioma
OS	Overall survival
PA	Pilocytic astrocytoma
PFS	Progression-free survival
RT	Radiotherapy
SAT	Systemic anticancer therapy
